# Adult Growth Hormone Deficiency – Benefits, Side Effects, and Risks of Growth Hormone Replacement

**DOI:** 10.3389/fendo.2013.00064

**Published:** 2013-06-04

**Authors:** Mary L. Reed, George R. Merriam, Atil Y. Kargi

**Affiliations:** ^1^Geriatrics and Extended Care, VA Puget Sound Health Care System, Madigan Health Care System, Tacoma, WA, USA; ^2^Division of Metabolism, Endocrinology, and Nutrition, VA Puget Sound Health Care System, University of Washington School of Medicine, Tacoma, WA, USA; ^3^Division of Endocrinology, Diabetes, and Metabolism, University of Miami Miller School of Medicine, Miami, FL, USA

**Keywords:** growth hormone, adult growth hormone deficiency, growth hormone risks, insulin-like growth factor-I, IGF-I, growth hormone treatment

## Abstract

Deficiency of growth hormone (GH) in adults results in a syndrome characterized by decreased muscle mass and exercise capacity, increased visceral fat, impaired quality of life, unfavorable alterations in lipid profile and markers of cardiovascular risk, decrease in bone mass and integrity, and increased mortality. When dosed appropriately, GH replacement therapy (GHRT) is well tolerated, with a low incidence of side effects, and improves most of the alterations observed in GH deficiency (GHD); beneficial effects on mortality, cardiovascular events, and fracture rates, however, remain to be conclusively demonstrated. The potential of GH to act as a mitogen has resulted in concern over the possibility of increased *de novo* tumors or recurrence of pre-existing malignancies in individuals treated with GH. Though studies of adults who received GHRT in childhood have produced conflicting reports in this regard, long-term surveillance of adult GHRT has not demonstrated increased cancer risk or mortality.

## Introduction

Adult growth hormone deficiency (AGHD) results in a clinical syndrome characterized by alterations in body composition, diminished aerobic exercise capacity and quality of life (QoL), and adverse changes in lipid and carbohydrate metabolism and cardiovascular function (Hazem et al., 2012). A growing body of evidence shows increased mortality in untreated AGHD patients (Rosén and Bengtsson, 1990). Treatment of AGHD patients with growth hormone replacement therapy (GHRT) normalizes many of these signs and symptoms, improves QoL, and may possibly enhance longevity (Rosén and Bengtsson, 1990; Hazem et al., 2012). Although a theoretical increased risk of developing new or recurrent neoplasms has been suggested in some studies evaluating adults who had been treated for GHD as children, this increase has not been found in most studies of treatment in patients with adult-onset GHD (Svensson and Bengtsson, 2009).

Identifying GHD in adults can be difficult due to similarities with normal senescence. Due to the lack of specific biomarkers for GHD, provocative biochemical testing is necessary to diagnose AGHD with adequate accuracy, but currently available tests are complex and may have significant side effects. A novel approach to diagnosis of AGHD has been the use of ghrelin mimetic GH secretagogues. These tests may offer a safe and reliable alternative to the insulin tolerance test (ITT) or the glucagon stimulation test (GST) for diagnosing AGHD.

Safety with long-term use and the cost effectiveness of GHRT have not been clearly determined in past studies. In addition, a growing incidence of acquired GHD related to traumatic brain injury (TBI) presents a new sub-population of GHD patients and consequently potentially increased numbers seeking treatment.

This review summarizes current understanding of the benefits and possible risks of GHRT, as well as recommendations for testing for GHD in adults, appropriate dosing, and monitoring for efficacy and cost effectiveness of GHRT.

## Background

Many of the clinical features of AGHD resemble aspects of normal aging. AGHD is a clinical syndrome associated with alterations in body composition with reduced bone and muscle mass, diminished exercise performance and cardiac capacity, and altered lipid metabolism with increase in adiposity (Kargi and Merriam, 2011). AGHD has also been associated with impaired cognition as well as having psychological impact (Deijen et al., 2011). All these features result in decreased QoL. The syndrome of AGHD may contribute to the increase in morbidity and mortality among patients with hypopituitarism.

## Incidence

Three nationwide registries in Denmark using uniform standardized criteria to diagnose adult-onset GHD estimated 19 cases per million for males and 14.2 cases per million for females, with an increasing incidence in age with males but not with females. Those individuals age 45 and older demonstrated the gender disparity not seen in younger patient populations. AGHD has been estimated to affect 1 per 100,000 people annually, while its incidence rate is approximately 2 per 100,000 when child-onset GHD individuals transition to adulthood (Stochholm et al., 2006).

## Prevalence and Etiology

Approximately 6,000 new cases of adults with GHD are diagnosed each year in the US. Some, 15–20% of those cases represent the continuation of child-onset GHD into maturity; the remainder have adult-onset GHD resulting from damage to the hypothalamic-pituitary axis (Gharib et al., 1998). Such damage most commonly arises from pituitary or peri-pituitary tumors, or by their treatment. Pituitary adenomas and their treatment account for nearly two-thirds of AGHD cases (Bates et al., 1996).

In addition to tumors and those adults who transition from child-onset GHD, a growing number of cases of GHD have been reported after TBI (Schneider et al., 2007). Most cases of GHD and other post-traumatic hypopituitarism (PTHP) have been diagnosed within 1 year of trauma, but there have been cases that developed nearly 40 years later (Edwards and Clark, 1986; Gunn et al., 1991) leading to speculation that unless patients were specifically questioned about past head trauma, many of these cases had been classified as idiopathic (Benvenga et al., 2000).

The mechanism of GHD or other hypopituitary disorders resulting from TBI is not clearly understood, but may reflect both hypothalamic and pituitary damage as well as disruption of hypothalamic-portal transport of growth hormone-releasing hormone (GHRH) and other regulatory peptides. It is thought that injury to the somatotrophs in the anterior pituitary, where GH is produced and released, may predispose these cells to anoxia, vascular insufficiency, or stalk injury. The long hypophysial vessels, which provide oxygen and nutrients to the anterior pituitary and the portal capillaries in the stalk, which transport hypothalamic regulatory peptides, are both highly vulnerable to traumatic injury, resulting in disrupted portal transport of GHRH or even anterior lobe infarction. Direct injury to the pituitary gland, resulting in edema of the diaphragma sella, can also result in anterior lobe infarction. Injuries to the hypothalamus, anterior pituitary, or pituitary stalk have been reported in 26–86% of patients who have died from TBI during autopsy (Daniel et al., 1959; Ceballos, 1966; Kornblum and Fisher, 1969; Crompton, 1971; Pierucci et al., 1971). In more recent studies, preliminary data suggests a possible association of certain apolipoprotein E polymorphisms with the development of TBI-induced pituitary dysfunction (Tanriverdi et al., 2008). The prevalence of post-traumatic AGHD can range from 9 to 28%; but this range may be severely underestimated, since many of studies have relied on insensitive biomarkers such as insulin growth factor-1 (IGF-1) to screen for GHD. This variation highlights the challenges for the accurate diagnosis of GHD in this sub-population (Bondanelli et al., 2004; Dimopoulou et al., 2004; Lieberman et al., 2008).

Traumatic injury is the leading cause of death and disability in young adults (Klasbeek et al., 1980; National Institutes of Health, 1998; van Baalen et al., 2003). It is estimated that 0.6–1.9 million dollars per person to over 5 million persons living with the sequelae of TBI is spent in overall healthcare costs and associated loss of productivity (McGregor and Pentland, 1997; Masel, 2004). TBI has been called the “signature injury” of the current wars in Southwest Asia. Over 10–20% of service members returning from a combat deployment have reported suffering at least one blast injury (Terrio et al., 2009). Fatigue, depression, irritability, cognitive impairment, and decreased QoL; symptoms associated with GHD, resemble another characteristic invisible injury of combat; post-traumatic stress disorder. As many as 25% of TBI survivors may go undiagnosed, which may contribute to long-term adverse consequences of untreated GHD. The implications of post-traumatic induced GHD demand a clear consensus for diagnosis, treatment, and management in this sub-population.

## Clinical Features of GHD

The function of several organ systems is directly altered by the loss of the physiologic effects of GH. Acquired isolated GHD is characterized by weight gain, increased fat mass, and decreased lean body mass (LBM). AGHD patients have 7% higher total body fat, with similarly decreased LBM (Cuneo et al., 1992; Carroll et al., 1998). Central adiposity lends to increased waist:hip ratio. In addition, triglyceride levels are increased and high density lipoprotein (HDL) levels decreased. This alteration in lipid levels may explain, in part, the observation of increased intima-medial wall thickness (IMT), as measured by carotid ultrasonography, in this population (Leonsson et al., 2002; Murata et al., 2003; Abs et al., 2006). These factors may contribute to the increased incidence of cardiovascular mortality seen in patients with GHD.

Decreased muscle mass and strength are also present in GHD patients. In the heart, these changes are manifested by reduction in left ventricular mass, decreased fractional shortening of cardiac myocytes, and decreased cardiac output. Such abnormalities may contribute to the decline in exercise capacity. Amato et al. (1993) demonstrated a reduction in exercise capacity by 20–25% in GHD patients compared to normal controls. In addition, cortical bone density and trabecular bone density measurements in GHD patients were 2.8 and 1.5 SD, respectively, below the mean age and sex matched controls (Amato et al., 1993; O’Halloran et al., 1993).

Patients with GHD appear to have impaired psychological well-being and potentially significant neuropsychiatric manifestations, such as difficulty concentrating and memory impairment (McGauley et al., 1990). The psychological general well-being (PGWB) scale was adapted by McKenna et al. (1999) to determine a measurable index of six dimensions of subjective well-being or distress. A version of the QoL-assessment of growth hormone deficiency in adults (QoL-AGHDA) questionnaire is shown in Table 1. Reduced vitality, fatigue, social isolation, and depression were consistently reported on self-rated questionnaires (Dupuy, 1984; McKenna et al., 1999). Other tools like the KIMS patient life situation form (KIMS PLSF) (Moock et al., 2009), Euro-QoL, EQ-5D (EuroQoL Group, 1990), and Nottingham health profile (NHP) (Hunt et al., 1981) have been developed to measure QoL and assess psychological well being (Hunt and McKenna, 1992; Saller et al., 2006; Koltowska-Häggström et al., 2009). It is unclear however whether this impairment in psychological well being is due specifically to reduced levels of GHD.

**Table 1:** **Adult growth hormone deficiency quality of life assessment (QoL-AGHDA) (reproduced from McKenna et al., 1999)**.

	Yes	No
I have to struggle to finish jobs		
I feel a strong need to sleep during the day		
I often feel lonely even when I am with other people		
I have to read things several times before they sink in		
It is difficult for me to make friends		
It takes a lot of effort for me to do simple tasks		
I have difficulty controlling my emotions		
I often lose track of what I want to say		
I lack confidence		
I have to push myself to do things		
I often feel very tense		
I feel as if I let people down		
I find it hard to mix with people		
I feel worn out even when I’ve not done anything		
There are times when I feel very low		
I avoid responsibility if possible		
I avoid mixing with people I don’t know well		
I feel as if I am a burden to people		
I often forget what people have said to me		
I find it difficult to plan ahead		
I am easily irritated by other people		
I often feel too tired to do the things I ought to do		
I have to force myself to do all the things that need doing		
I often have to force myself to stay awake		
My memory lets me down		

Instructions: indicate whether each of the following statements below applies to you. Scoring: 1 point for each “yes” answer.Used with permission from Pfizer Inc.

The signs and symptoms of GHD are summarized in Table 2 (Cuneo et al., 1992; Carroll et al., 1998).

**Table 2:** **The syndrome of adult growth hormone deficiency (Cuneo et al., 1992; Carroll et al., 1998)**.

Increased fat mass (especially central adiposity)
Decreased lean body mass
Decreased muscle strength
Decreased exercise performance
Decreased cardiac capacity
Decreased bone mineral density and increased risk of fracture
Atherogenic lipid profile
Thin, dry skin
Psychosocial problems and decreased quality of life
Fatigue
Depression
Anxiety
Impaired sleep
Social isolation

## Diagnosis of GHD

Given the lack of specific clinical marker for GHD in adults, biochemical confirmation of the diagnosis is essential and usually entails a stimulation test with a substance that triggers a rise in GH in normals. Because the specificity of all available tests is imperfect, testing should be reserved for those patients whose clinical presentation would yield a high pre-test probability of GHD, or if there is evidence of other pituitary-dependent hormones that would put them at risk of GHD, in order to decrease the rate of false positives.

Testing is indicated for patients with structural disease affecting the hypothalamus or pituitary, history of prior surgery to these areas, history of head trauma or subarachnoid hemorrhage, or documented deficiencies in other pituitary hormones (Cook et al., 2009; Molitch et al., 2011). Low levels of insulin-like growth factor-I (IGF-I) levels can be diagnostic in severe GHD, but since IGF-I is often normal in AGHD, it is often necessary to confirm diagnosis with provocative testing (Kargi and Merriam, 2013).

Hartman et al. (2002) reported that total IGF-I concentrations less than 84 mcg/L (11 nmol/L) in the presence of three or more pituitary hormone deficiencies the diagnosis of GHD can be made with 95% accuracy. IGF-I bioactivity however may be even more sensitive than total IGF-I levels, demonstrating a CI of 81.9 versus 63.8% in GHD patients (Varewijck et al., 2011). With these notable exceptions, provocative testing is still warranted in that nearly 58.5% of severely GHD patient have total IGF-I levels within age-related normal range (Cianfarani et al., 2005). Age, gender, and obesity interplay in IGF-I concentrations in GHD patients. There is an overlap of age-related decline in IGF-I levels in normal subjects and GHD patients with increasing age. GHD patients demonstrated 4% (*n* = 48), 30% (*n* = 61), and 40% (*n* = 43) IGF-I levels within the normal range limits for 20–39, 40–59, and over 60 years of age, respectively (Hilding et al., 1999). Prior reports have shown a much higher percentage of overlap, with range 34.8, 60.8, and 84.5% total IGF-I levels above the age-related third percentile limits for the same age ranges (Cianfarani et al., 2005). IGF-I levels are generally lower in female GHD patients versus male counterparts, but the exact mechanism is unclear and warrants further investigation (Fisker et al., 1999).

The ITT is still considered the “gold standard” diagnostic test for GHD, and should be considered as the initial test, unless there are contraindications to its use. Because it induces hypoglycemia, it is contraindicated in patients with coronary artery disease, seizures, and in the elderly (Biller et al., 2002).

The combination of GHRH with arginine (GHRH + ARG) was endorsed by several consensus guidelines as a comparably accurate alternative to ITT, especially when ITT is contraindicated (Aimaretti et al., 1998; Ho and Participants, 2007; Yuen et al., 2009). However, in 2008 EMD Serono, Inc. discontinued the manufacture of GHRH (Geref^®^) in the US. The unavailability of GHRH increased the need for identifying reliable alternative GH stimulation tests to diagnose AGHD with similar accuracy when ITT is contraindicated or logistically difficult to perform.

Several studies have since shown that the GST is as reliable as the ITT in discriminating GH-deficient and normal adults (Spathis et al., 1974; Conceição et al., 2003; Yuen et al., 2009). Gomez et al. and Conceicao et al. demonstrated that the glucagon test reliably identified controls and GHD patients. Using a peak GH level of 3 ng/mL as a cutoff provided the best sensitivity (100 and 97%, respectively) and specificity (100 and 88%, respectively) by receiver-operating characteristic (ROC) curve analysis (Gómez et al., 2002; Conceição et al., 2003; Berg et al., 2010; Yuen, 2011). The study of Berg et al. (2010) reported somewhat lower accuracy – 95% sensitivity and 79% specificity with a cutoff set at 2.5 ng/mL, most likely reflecting a difference in the subject population tested.

Growth hormone secretagogues, such as ghrelin and ghrelin mimetics (hexarelin, macimorelin, and GH-releasing peptide 2 and 6 – all currently still investigational) have also been studied for use in diagnosing AGHD. With macimorelin, an orally active GH secretagogue, a recent study reported that peak GH (an optimal cut point of 2.7 ng/mL) offered 82% sensitivity, 92% specificity, and 87% accuracy (Garcia et al., 2013). In general macimorelin was well tolerated; and compared to other stimulation tests, GH responses were rapid and the test does not require parental administration of agents.

Consensus guidelines for the evaluation of adult GHD state that any of these dynamic tests can be used to diagnose GHD, including the ITT, the GST, the combined GHRH–ARG test (where available), and likely the ghrelin mimetics when further validated. Age, gender, and BMI may blunt the response of some stimulatory tests. Further investigation is warranted in determining the best cut off values to enhance the sensitivity in diagnosing GHD in this subset population (Colao et al., 2009). Table 3 provides a comparison chart for GH provocative tests.

**Table 3:** **Comparison of GH Provocative Tests (Kargi and Merriam, 2013)**.

Test	Protocol	GH diagnostic cutoff	Advantages	Additional comments
Insulin-induced hypoglycemia (ITT)	Regular insulin 0.1 units/kg IV Measure glucose, GH, and cortisol at baseline and every 30 min for 3 h	5 ng/mL	Gold standard test Simultaneously assesses HPA axis	Risk of serious side effects Significant contraindications Resource intensive
GHRH-Arg	GHRH 1 μg/kg Arginine 0.5 g/kg over 30 min Measure GH at baseline and every 30 min for 3 h	BMI <25: 11 ng/mL BMI 25–30: 8 ng/mL BMI >30: 4 ng/mL	Low risk of side effects	GHRH not commercially available in US Low sensitivity for GH diagnosis in patients with hypothalamic etiologies of GHD
Glucagon stimulation test (GST)	Glucagon 1 mg i.m. or s.c. (1.5 mg for patients >90 kg), not i.v. Measure glucose and GH (and cortisol if desired) at baseline and every 30 min for 4 h	3 ng/mL	Glucagon readily available	Side effects (nausea, vomiting, headache)
GH secretagogues (ghrelin and ghrelin mimetics)	1 mcg/kg IV Measure GH response every 15 min for 120 min	BMI <25: 7.3 ng/mL) BMI 25–30: 2.9 BMI >30: 0.6	GH peak in 60 min	Oral formulation; side effect – bad taste (macimorelin)

## Treatment of GHD; Benefits of GHRT

There is a general consensus that many of the metabolic and psychological abnormalities associated with GHD can be reversed with GH replacement. Lower doses of GH may decrease the incidence of side effects from GHRT, while still achieving a clinical response to therapy (Chein et al., 1999; Ahmad et al., 2001). Treatment with GH results in profound changes in body composition, especially in reduced fat mass, and an increase in LBM. The decline in fat mass is most significant in visceral and trunk locations (9% reduction, compared with placebo-treated patients who have a mean 4.3% increase in visceral fat mass over 6 months), suggesting that GHRT may reverse the central adiposity associated with GHD and potentially reduce the increased cardiovascular risk that this body composition carries (Beauregard et al., 2008). The increase in LBM is smaller than the reduction in fat mass, but appears to be more sustained (Al-Shoumer et al., 1996; Hoffman et al., 2004a; Götherström et al., 2009).

An overall beneficial effect on serum lipid profile (reduction in total and LDL cholesterol and improvement in HDL cholesterol) has been noted with GHRT in some studies, but data are inconsistent (Florakis et al., 2000; Maison et al., 2004). When GHRT is added to lipid management therapy with HMG CoA reductase inhibitors (statins), it may promote a synergistic effect (Monson et al., 2007). The reduction in atherosclerosis with use of GHRT has been assessed by several studies evaluating carotid IMT (Pfeifer et al., 1999; Leonsson et al., 2002; Murata et al., 2003; Colao et al., 2008). In addition, GHRT has demonstrated improvement in pro-inflammatory and other cardiovascular risk markers, including C-reactive protein, apolipoprotein B, and homocysteine levels (Sesmilo et al., 2000, 2001).

Growth hormone replacement has a positive effect on left ventricular mass, interventricular septal and left ventricular posterior wall thickness, left ventricular ejection diastolic diameters, and stroke volume, via echocardiographic evaluation in adults with GHD (Maison and Chanson, 2003). It improves both exercise capacity and cardiac function (Widdowson and Gibney, 2008). These patients demonstrated increased oxygen uptake and power output with cycle ergometry with increased skeletal muscle mass and aerobic capacity.

Growth hormone replacement therapy results in a biphasic change in bone mineral density (BMD), with an initial 6–12 months period of increased bone resorption followed by an overall increase in bone mass. After long-term treatment, BMD continues to rise even 18–24 months after discontinuation of GHRT (Baum et al., 1996; Johannsson et al., 1996; Biller et al., 2000). Typically a 4–10% increase in BMD has been reported, with generally greater effects in vertebral trabecular bone compared to femoral sites (Baum et al., 1996; Biller et al., 2000; Shalet et al., 2003). In general individuals with the most severe bone loss had the greatest positive response to treatment, and BMD in men responded to a greater degree than BMD in women, perhaps reflecting estrogen-mediated inhibition of GH action through stimulation of suppressor of cytokine signaling-2 (socs2) (Johannsson et al., 1996; Drake et al., 2001; Bex et al., 2002).

There appears to be no single factor that completely accounts for the increased mortality seen in untreated GHD adults when compared to age and sex matched controls (Rosén and Bengtsson, 1990; Hazem et al., 2012). The two most prominent causes of premature mortality in GHD patients are cardiovascular and cerebrovascular disease. Despite several studies of the effects of chronic GH replacement on mortality, the evidence of increased longevity is still not strong enough to be conclusive (van Bunderen et al., 2011; Gaillard et al., 2012).

The majority of GHD patients report enhanced QoL after starting GHRT as assessed both by self-rating questionnaires and by objective measures such as days absent from work and economic success. The severity of QoL impairment before therapy with GH is the strongest predictor of treatment response (Murray et al., 1999). QoL measures improve as soon as 3 months after initiating GHRT, and long-term studies shows a continued benefit for 10 years (Drake et al., 1998; Gilchrist et al., 2002; Rosilio et al., 2004).

## Indications for GH Replacement

Prior to 1996 the use of GH in the US was limited to promoting statural growth in children with confirmed GHD; its use to promote growth in other conditions associated with short stature, such as Turner syndrome, was approved subsequently. It has only been in the past 20 years that compelling evidence for a clinical syndrome in adults with GHD has been published and widely accepted. While several consensus guidelines have been published on the management of AGHD with GH replacement, the optimal criteria to select patients who might best benefit from treatment remains controversial, and practices vary among individual practitioners and countries.

In general it is recommended that only adults with confirmed GHD based on published criteria be evaluated for treatment. Individuals most likely to benefit from GH replacement include those with severe GHD, defined by GH provocative testing response and serum IGF-I levels. Other appropriate indications for initiation of GHRT are osteopenia, adverse cardiovascular risk profile, and reduced QoL, as measured from validated QoL questionnaires. Continuation of therapy is generally recommended for adolescents transitioning to adulthood even after completion of linear growth, to allow full skeletal and muscle maturation, which can continue for a further decade (Bex et al., 2002; Baroncelli et al., 2003; Shalet et al., 2003; Underwood et al., 2003). Discontinuation of GHRT based on reaching final adult height in this transitioning population of GHD patients has also been associated with worsening of lipoprotein profiles, body composition, and QoL scores (Underwood et al., 2003; Mauras et al., 2005) GH therapy in this population should therefore be interrupted as briefly as possible, optimally only for re-testing to confirm persistent GHD.

## Initiation and Monitoring of GH Treatment

Unlike pediatric GH treatment, often dosed in micrograms/kilograms of body weight/day, currently recommended GH replacement dosing in adults is individualized independent of weight, taking into account the patient’s age, gender, and estrogen status (Johannsson et al., 1997a; Hoffman et al., 2004b). Initiating therapy at low doses (total dose 0.2–0.4 mg/day SC) decreases the likelihood of developing common side effects like joint stiffness, arthralgias, myalgias, paresthesias, and peripheral edema, with fluid retention. The dose should be titrated at 6–8 week intervals based on clinical response, while avoiding side effects and monitoring serum IGF-I levels. Achieving a target in the upper half of normal age adjusted IGF-I range is desired. It is reasonable to start with higher doses (0.4–0.5 mg/day) in patients less than 30 years of age, but older patients (greater than 60 years of age) should be started on lower doses (0.1–0.2 mg/day) and titrated more slowly to minimize occurrence of side effects. Some childhood-onset AGHD patients with very low pre-treatment IGF-I may develop GH side effects but still not reach mid-normal levels despite high GH doses.

Women taking oral estrogen replacement may require higher doses of GHRT, presumably because oral estrogen inhibits liver IGF-I production and secretion by a first pass effect (Weissberger et al., 1991). No dose adjustment is usually needed in patients on moderate doses of transdermal estrogen.

Once dosing is stabilized, clinical evaluation and assessment for side effects along with measurement of IGF-I levels should be performed at 6 month intervals. Fasting lipid profiles and fasting glucose levels should be checked annually and after any GH dose increase. Blood pressure, weight, waist circumference, and BMI should be checked annually and at each clinic visit. Baseline bone DEXA scan should be performed and then rechecked every 2 years if the baseline is abnormal. Optimally QoL measures should be assessed at baseline and then at least annually while on GHRT to monitor treatment response (Cook et al., 2009; Koltowska-Häggström et al., 2009).

The duration of treatment for AGHD has not been defined. If therapy is tolerated, with a good clinical response to treatment noted, then there is no particular reason to discontinue therapy. Conversely if there is no perceived or biochemical benefit of treatment after at least 1 year of therapy, then stopping GHRT may be appropriate.

Tables 4 and 5 summarize the 2009 AACE recommendations for GHRT dosing and monitoring (Cook et al., 2009).

**Table 4:** **Factors that may affect GH dosing [adapted from AACE 2009 (Cook et al., 2009)]**.

Increase GH dose	Decrease GH dose
Younger patients (regardless of onset type)	Older patients
Low IGF-I levels	High IGF-I levels
Oral estrogen use	Discontinuing oral estrogen/change to transdermal estrogen use
To induce lipolysis	Testosterone use (not always necessary}
	Worsening glucose tolerance
	Side effects

Reprinted from endocrine practice, vol. 15 (Cook et al., 2009), with permission from the American Association of Clinical Endocrinologists.

**Table 5:** **Recommendations for GH replacement therapy in AGHD (adapted from Cook et al., 2009)**.

**Starting dose**
Age <30 years: 0.4–0.5 mg/day (may be higher for patients transitioning from pediatric treatment)
Age 30–60 years: 0.2–0.3 mg/day
Age >60 years: 0.1–0.2 mg/day
Use lower GH doses (0.1–0.2 mg/day) in all patients with diabetes or who are susceptible to glucose intolerance
**Dose titration**
At 1–2 month intervals, increase dose by 0.1–0.2 mg/day based on clinical response, serum IGF-I levels, side effects, and individual considerations such as glucose intolerance
In older patients consider longer time intervals and smaller dose increments
**Goal**
Aim for serum IGF-I levels in the middle of the normal range appropriate for age and sex, unless side effects are significant
Consider a trial of higher GH doses to determine additional benefit as long as serum IGF-I levels remain within normal range and no adverse side effects reported

Reprinted from endocrine practice, vol. 15 (Cook et al., 2009), with permission from the American Association of Clinical Endocrinologists.

## Novel GH Formulations and Analogs

Traditional treatment of GHD has been with the use of daily subcutaneous injections of recombinant human growth hormone (rhGH). This schedule however, is too infrequent to reproduce the multiple daily pulses of normal endogenous GH secretion, and too frequent to be convenient for many patients, raising concerns about adherence to treatment, which can lead to reduced efficacy. Although rhGH when injected SC has a circulating half-life of 5 h and is rapidly cleared by the liver and kidney, studies have demonstrated comparable growth velocity and IGF-I levels with continuous infusion pump therapy (Jørgensen et al., 1990; Laursen et al., 1993, 1995, 2001; Tauber et al., 1993). This suggests that longer-acting GH formulations and analogs, although even more unphysiologic than daily injections, could be effective for AGHD treatment.

A number of brands and formulations of rhGH are available; in the US, GH preparations are available as lyophilized powder in vials for reconstitution (Humatrope^®^, Nutropin^®^, Serostim^®^, Saizen^®^, Zorbtive^®^, Tev-Tropin^®^, and Omnitrope^®^); in two chamber cartridges requiring reconstitution (Genotropin^®^ and Saizen^®^); as a liquid in prefilled cartridges (Norditropin^®^, Humatrope^®^, Nutropin AQ^®^, Saizen^®^, and Omnitrope^®^); or as prefilled single-dose (Genotropin Miniquick^®^) or multi-dose pens (e.g., Norditropin^®^ Flexpro^®^, Norditropin^®^, Nordi-Flex^®^, Nutropin AQ^®^ – Nuspin™, or Omnitrope^®^ 5 or 10 mg pens). Those with preservative-free diluents must be used within 24 h of reconstitution, but others can be stored unrefrigerated for 14–28 days (Nutropin, 2006, 2008; Saizen, 2007; Serostim, 2007; Tev-Tropin, 2007; Genotropin, 2009; Humatrope, 2009; Zorbtive, 2009; Norditropin, 2010; Omnitrope, 2010).

Besides daily GH, several longer-acting GH formulations and analogs have been developed. The most fully studied sustained-release formulation thus far is encapsulation of GH into biodegradable polylactic acid-co-glycolic acid (PLGA) microspheres, which showed sustained GH release profile for 1 month (Johnson et al., 1997; Park et al., 2010). Nutropin Depot^®^ was the first GH delivery system launched using the PLGA microsphere system, but was later taken off the market due to the high injection volumes and large-bore needles required, along with business concerns over the manufacture (Govardhan et al., 2005).

A GH-loaded hyaluronate microparticle (LB03002) was developed and launched in Korea by LG Life Sciences in 2007. This once a-week injection is currently the single commercially available sustained-release formulation of GH, at present approved only in that country. In global Phase III study, treatment with LB03002 for 26 or 52 weeks in adults with GHD demonstrated a sustained reduction of fat mass and other body composition parameters, and was generally well tolerated with a good safety profile (Biller et al., 2010; Péter et al., 2012).

PEGylation (the attachment of polyethylene glycol, PEG) has also been used to extend the half-life of rhGH. The first PEGylated rhGH (PHA-794428) was discontinued due to safety concerns related to local injection-site lipoatrophy (Touraine et al., 2009). Two other longer-acting GH extension peptides using either albumin or an hCG-derived extension sequence are also in development.

VRS-317 is a novel rhGH fusion protein (addition of hormonally inert and relatively non-allergenic N- and C-terminal amino acids extend the half-life of rhGH) that may provide a monthly dosing option in GHD patients. In animal subjects, VRS-317 had a half-life of approximately 110 h, and produced a sustained IGF-I response for 1 month after a single dose. A Phase I trial of VRS-317 in GHD adults has recently been completed. Preliminary data show achievement of graded responses of IGF-1 at doses lower than those typically used with daily rhGH, with no significant drug related adverse events reported over the course of 1 month (Bailey et al., 2012; Cleland et al., 2012). If these early results are confirmed in multi-dose studies, monthly dosing could lead to increased adherence and convenience.

## Side Effects and Risks, and Long-Term Safety Concerns

The most common side effects of GH treatment in GHD adults result from fluid retention, with peripheral edema, arthralgias, carpal tunnel syndrome, paresthesias, and worsening of glucose tolerance. These hormonal side effects generally respond to dose reduction. Older and more obese patients are more susceptible to side effects from GH treatment. In studies using higher than recommended doses of GH, more frequent adverse outcomes have been observed (Johannsson et al., 1997a; Hoffman et al., 2004b).

Benign intracranial hypertension (pseudotumor cerebri) has been linked to GH treatment in children (Malozowski and Stadel, 1995) but is rare in adults, with only one case reported (Malozowski et al., 1993). Gynecomastia has been reported in normal elderly individuals receiving GH in high doses (Cohn et al., 1993; Blackman et al., 2002).

Another rare but reported complication of GH therapy is macular edema in non-diabetic patients. Koller et al. (1998) reported two non-diabetic patients: one a non-obese 11-year-old girl receiving GHRT for Turner’s syndrome presenting with neovascularization, and an obese 31-year-old male who had traumatic hypothalamic injury and was started on GHRT 14 months prior to presenting with decreased visual acuity. Of note, both cases had their GH dose increased just prior to the onset of this complication.

Early clinical trials reported insulin resistance and diabetes mellitus in patients receiving GHRT. These small initial studies reported impaired fasting glucose and insulin levels within the first year of GH therapy (Beshyah et al., 1994; Salomon et al., 1994; al-Shoumer et al., 1998). In addition, data from the Pfizer International Growth Database (KIGS) indicate that GH therapy in children may give rise to an increased incidence of type 2 diabetes mellitus, heightening the concern for adults with GHD receiving similar treatment (Cutfield et al., 2000). Many of the early AGHD studies used weight-based dosing derived from pediatric practice, and these fluid retention and hyperglycemic side effects have become much less frequent as adult practice has shifted to individualized dosing starting with lower doses and gradually titrating upwards until target responses, benefits, or side effects are encountered. Perhaps for this reason, long-term GH replacement in adult patients with GHD has not usually led to abnormal levels of plasma glucose or glycosylated hemoglobin (Bengtsson et al., 2002). In fact, some studies of titrated GH replacement for AGHD have been shown to improve insulin sensitivity, at least in patients with features consistent with metabolic syndrome (Johannsson et al., 1997b), suggesting that improved body composition can at least partially counter the direct anti-insulin actions of GH, although in non-obese subjects the net effect of GH treatment is usually a mild increase in fasting glucose and/or insulin within the normal range. Insulin sensitivity varies based on the patient’s genetic predisposition, age, and body composition (Hoffman et al., 2004a). Diabetes is not an absolute contraindication to GHRT, but in individuals with diabetes or predisposed to developing diabetes, it is recommended to titrate the dose of GH even more slowly than in other patients, and to adjust anti-diabetic medications as warranted to maintain glucose homeostasis.

The generally benign side effect profile of replacement doses of GH cannot be extrapolated to supraphysiologic dosing intended to overcome GH resistance or to reverse catabolism. Takala et al. (1999) reported increased mortality in parallel studies conducted on critically ill patients treated with much higher doses of GH (5.3–8 mg/day), more than 10-fold the doses often used in adult GH replacement. Reasoning that these patients had resistance to GH based on high GH levels but low serum IGF-I and IGF-binding protein-3 concentrations, due to increased protein catabolism and negative nitrogen balance, these investigators attempted to reverse this catabolic state; but instead found a near-doubling of the already high in-hospital death rate, though with no single mechanism to which the increased mortality could be attributed. Non-fatal fluid retention, which can be significant, is also more commonly associated with high GH doses, particularly in older patients (70% in Finnish study were 55 years of age and older and 75% in the multinational study) (Johannsson et al., 1997a; Hoffman et al., 2004b).

There is no evidence that GH replacement in adults increases the risk of *de novo* or recurrent malignancy. For survivors of childhood cancer, GH treatment may further slightly increase the already increased risk of developing a second neoplasm, although this increase was not seen in the large Childhood Cancer Survival Study (Sklar et al., 2002). However, there are no comparable data for adult GHRT. Most of the concerns about increased cancer rates in GHD patients treated with GH have primarily focused on observational data on survivors of childhood leukemia, in whom cranial irradiation frequently leads to GHD. These reports may be misleading however, since it is not clear if tumor development noted reflected a new or recurrent malignancy, or due to irradiation or other past treatment of existing tumors (Swerdlow et al., 2002). Notably, pediatric patients with “idiopathic” GHD did not have an increase in new tumor growth when treated with GH (Fradkin et al., 1993).

Despite the overall lack of a signal for increased risk, case reports and a minority of series have noted development of cancers after treatment with GH. Magnavita et al. (1996) reported a case of a semi-professional cyclist who developed Hodgkin’s lymphoma nearly 4 years after several supra-therapeutic courses of GH. Swerdlow et al. (2002) reported an increased incidence of cancer formation in a cohort study of 1,848 patients in the UK treated with GH from 1959 to 1985. Cancer incidence was assessed in 1995 and again in 2000. Despite the author’s assertion that there was a high incidence of cancer and mortality associated with cancer, the total number of cancer diagnoses in the cohort was only 12 – 2 colon cancer, 2 Hodgkin’s lymphoma, 2 bone cancer, and 1 each from mouth, liver, bile duct, cervix, ovary, and testis. Only the first two increases were statistically significant. Of note, one patient who developed colon cancer may have had familial polyposis. Despite the small number of cancer cases, there is an epidemiological association of cancer rates in the setting of increased IGF-I levels but not of IGF-binding protein-3 (IGFBP-3), resulting in a high IGF-1 to IGFBP-3 ratio (Grimberg and Cohen, 1999; Shim and Cohen, 1999; Cohen et al., 2000). IGF-1 has been detected in colorectal cancers and is a strong stimulator of colorectal cancer cell proliferation *in vitro* (Lahm et al., 1994), but GH treatment usually results in increases in both IGF-I and IGFBP-3. There is no information on IGF-1 and IGFBP-3 measurements in the Swerdlow report. The British patients in this cohort were given high GH doses 2–3 times per week versus current use of lower daily dosing, and this difference may also have played a role in these findings. Data are currently lacking demonstrating whether current GH dosing regimens promote any tumor recurrence or re-growth (Frajese et al., 2001; Hatrick et al., 2002; Chung et al., 2005, 2008; Jostel et al., 2005; Karavitaki et al., 2006; Buchfelder et al., 2007; Arnold et al., 2009; Olsson et al., 2009).

Growth hormone is a mitogen, however, and despite several studies that show no demonstrated increased risk of malignancy with GHRT, the use of GH is contraindicated in active malignancy out of concern it might accelerate the growth of an existing neoplasm (Frajese et al., 2001; Hatrick et al., 2002; Chung et al., 2005, 2008; Jostel et al., 2005; Karavitaki et al., 2006; Buchfelder et al., 2007; Arnold et al., 2009; Olsson et al., 2009). GH therapy should be stopped in all patients with active malignancy until the underlying condition is controlled.

## Use of GH in Non-GHD Adults: Normal Aging and Competitive Sports

### Aging

After achieving linear growth and full reproductive maturation, GH levels begin to decline; primarily from reduced hypothalamic secretion of GH-releasing hormone, which leads to lower GH levels and reduction in serum IGF-I levels. These normal age-related GH and serum IGF-I reductions are associated with age-related changes that are similar to the signs and symptoms seen in GHD adults. Based on this decline and in alterations in body composition, strength, and aerobic capacity that are also similar to those observed in AGHD, though less severe, interest was piqued in using GH therapy in healthy older patients. In 1990, Rudman et al. (1990) reported reduced fat mass, increased muscle mass and increased lumbar vertebral bone density in healthy men, age 60 and older, after 6 months of GH use compared to untreated controls. Although no functional outcomes were reported, this paper received wide publicity and led to subsequent studies looking to confirm the possible anti-aging benefits of GH in healthy seniors, as well as to rapid proliferation of anti-aging clinics offering GH to middle-aged as well as older clients, even in the absence of any confirmatory results.

Liu et al. conducted a systematic review of 31 articles looking at 18 separate studies of GH treatment outcomes in healthy older individuals. They concluded that only minimal body composition changes (2.1 kg reduction in fat mass, 2.1 kg increase in LBM, and 0.29 mmol/L reduction in total cholesterol) without significant change in bone density, other serum lipid levels or decrease in body weight was seen with GH use. However, a higher number of adverse events (edema, arthralgias, gynecomastia, development of impaired fasting glucose, and diabetes) were reported (Liu et al., 2007).

Blackman et al. (2002) evaluated effects of GH and/or sex steroid treatment in older men and women in a 2-by-2 placebo-controlled study. They reported that GH alone and GH with testosterone similarly resulted in reduced fat mass and increased LBM. Testosterone alone or the combination of GH with testosterone improved strength and exercise capacity whereas GH use alone did not. Adverse effects were similar with and without sex steroids; except for a higher rate of fasting glucose intolerance or diabetes in men treated with GH only. These investigators initially attempted to use GH doses similar to those employed by Rudman and colleagues (Rudman et al., 1990; Blackman et al., 2002), but were forced to reduce them by nearly 2/3 due to severe side effects. The reasons for the lack of side effects in the Rudman report are not clear.

Reports of effects of GH on BMD in non-GHD normal aging are conflicting. Holloway et al. (1997) found statistically significant improvement in BMD in postmenopausal women treated with GH, but concluded that the gains were substantially less than what is seen with bisphosphonates or estrogenic hormone therapy, with a higher incidence of adverse events. However, other studies did not demonstrate BMD increase with GH treatment in this population (Gurlek and Gedik, 2001; Christmas et al., 2002).

Metformin and GH did not appear to be superior to metformin alone in reducing total body fat or waist circumference in older patients with metabolic syndrome and elevated fasting plasma glucose levels (Herrmann et al., 2004).

In a placebo-controlled study, Baker et al. (2012) demonstrated favorable effects on cognition in both healthy older adults and those with amnestic minimal cognitive impairment (MCI) with 20 weeks of daily self-injection with a degradation-protected GHRH analog to boost GH secretion. Treatment with GHRH, which boosted GH secretion and IGF-I levels resulted in improvements in executive functioning and verbal memory. Visual memory was not enhanced. Though GHRH was generally well tolerated, subjects on active treatment were twice as likely to have adverse effects, usually mild, compared to those in the placebo arm.

Thus the use of GH to counter some effects of normal aging is still highly controversial. Most studies have shown some improvements in body composition, but failed to show an increase in cardiovascular endurance or muscle strength (Blackman et al., 2002; Liu et al., 2007). Additionally more adverse events were reported in the GH treatment groups. Given that both the baseline and target differ from AGHD, with baseline levels that although lower than in young normals are higher than in GHD, and target levels above age-matched normals and similar to those in healthy young adults. It is unclear if the mix of benefits, side effects, and risks will be similar to those in AGHD, particularly with use over longer durations than the maximum 1 year in current controlled studies. Thus GH treatment in otherwise healthy seniors cannot be recommended other than in controlled clinical research studies, and its use for anti-aging purposes is currently prohibited by US Federal law, 21 U.S.C. §333(e), making GH the only legal drug for which off-label prescribing is illegal in two circumstances. The other prohibition is for use to boost athletic performance.

### Athletics

Anecdotal evidence suggests that GH and IGF-I are frequently abused by athletes for their anabolic and lipolytic properties. GH has been touted to achieve faster recovery from injury and enhance ergogenicity, although there is no evidence that GH or IGF-I actually improves competitive performance in young healthy adults (Healy and Russell-Jones, 1997; Holt and Sonksen, 2008; Liu et al., 2008; Baumann, 2012). Conducting randomized controlled clinical trials is challenging, due to frequent concomitant use of insulin and anabolic steroids. GH and IGF-I appear on many lists of prohibited substances in competition, and their use is banned by the World Anti-doping Agency (WADA). As noted, in the US, administration of GH (but not IGF-I, GHRH, or ghrelin mimetic GH secretagogues) to enhance athletic performance is legally prohibited, though rarely prosecuted; legal proceedings have generally been based on false testimony about its use rather than the use itself. GH may be appealing to athletes seeking an edge; given its short half-life in circulation its abuse is difficult to detect except in extremely high doses (Baumann, 2012). Increased IGF-I stimulated by GH self-administration does not qualitatively differ from that which is supported by endogenous GH. Although synthetic GH contains just one molecular weight isoform, while endogenous GH secretion is a mix of 20 and 22 kD isoforms, it is rapidly cleared from the circulation and so testing needs to be done within just hours after dosing (Baumann, 2012). Liu et al. (2008) conducted a systematic review of 27 randomized, controlled trials involving 303 young, lean, physically fit subjects receiving GH at an average dose of 2.5 mg/day, a 5- to 10-fold excess over the physiological GH production rate. As expected, body composition changes (increase LBM and reduced fat mass) were noted, but there were no differences in strength or exercise capacity between those taking GH and those not. One of the limitations he noted in these studies was the shorter duration of use and lower dose of GH compared to what is typically abused by athletes. The typical side effects associated with GH overdose (edema, arthralgias, carpal tunnel syndrome, and sweating) were observed in 15–44% of the participants (Holt and Sonksen, 2008).

## Cost Effectiveness

Managed care payers should utilize evidence-based review to determine appropriate criteria for reimbursement for GH therapy. Despite the positive effects of GH replacement treatment on body composition and markers of cardiovascular disease in adults with GHD, it is difficult to measure the therapeutic benefits of GHRT with precision after linear growth has been completed. In addition, despite multiple studies, data showing that GHRT in GHD adults actually reduces mortality are still inconclusive (Frajese et al., 2001). With the lack of other effective treatment outcome measures, the use of “softer” endpoints such as QoL, have been used to justify the cost effectiveness of GHRT.

Using quality adjusted life years (QALY) measures, a cost-utility analysis of GHRT may be feasible. QALY, a frequently used health status index, combines information on the length of life (quantity) and the QoL, where the latter is measured by utilities on a scale that has values of 1 and 0 for full health and death, respectively (Torrance, 1987). The QALY unit is therefore defined as 1 year of life with full health.

Cost-utility analysis based on QALY change is the most widely recognized method in pharmacoeconomic evaluation, and QoL-AGHDA was used by the UK National Institute for Health and Clinical Excellence (NICE) as a potential source of outcomes data for such an evaluation (McKenna et al., 1999). The NICE Committee agreed that the QoL-AGHDA questionnaire is the best validated available evaluation tool for the assessment of both baseline QoL and the effect of treatment in adults with GHD (Table 1), and concluded that a trial of GH treatment could be recommended for adults with GHD who have perceived severe impairment of QoL, as demonstrated by a reported score of at least 11 in the QoL-AGHDA. Upon examination of available evidence, the Committee found that the subgroup of adults with GHD for whom treatment may be clinically justified are those who have an improvement in QoL equivalent to an absolute change of at least seven points over their baseline score. The Committee stated that re-assessment of the need for GH replacement should take place after a trial treatment period of at least 9 months (3 months for dose titration and 6 months for assessment of response). For GH treatment to continue after this trial period, they recommended that one demonstrate a sustained improvement in QoL over the pre-treatment baseline. These recommendations are widely used in determining coverage for GH for patients covered by the National Health Service, but less universally adopted outside the UK.

In the US, many insurers have developed policies that set specific criteria for approved indications for coverage of GH replacement in AGHD and to guide clinicians with appropriate dosing, monitoring, and assessing cost effectiveness of GHRT. Some are evidence-based and follow the recommendations of the 2009 AACE or the 2011 Endocrine Society clinical practice guidelines but others are arbitrary and in some cases at variance with best practices (Cook et al., 2009; Molitch et al., 2011).

## Conclusion

Despite absence of the prominent biological marker of linear growth, AGHD has characteristic, if non-specific signs and symptoms. Many of these can be reversed with appropriate GHRT. Determining who should be tested and treated for GHD can be challenging with the relative insensitivity of simple available biochemical markers such as IGF-I. And current dynamic testing procedures are either complex or attended with significant side effects or risks, or both. Reliable and safe alternative diagnostic tools are therefore needed. Stimulation testing using ghrelin mimetics appears promising but is not yet clinically available. Research is ongoing in developing longer-acting and therefore more convenient preparations of GHRT to promote better compliance and clinical efficacy. It appears at this time that cost-benefit and cost effectiveness data for GHRT favors treatment in clinically symptomatic patients with confirmed GHD. Despite the validated beneficial effects of GHRT in adults – including improvements in body composition, lipid profile, QoL and BMD – shown in many studies, some controversies remain with regards to whether long-term use of GH might promote tumor initiation or recurrence.

## Conflict of Interest Statement

George R. Merriam has received research grants from Aeterna Zentaris, Lilly, and Versartis and has consulted for Novo Nordisk, Teva, and Theratechnologies. Mary L. Reed and Atil Y. Kargi declare no outside financial interests. The views expressed are those of the authors and do not reflect the official policy of the Department of the Army, the Department of Defense, or the US Government.
